# *SNCA* rs3910105 Is Associated With Development of Rapid Eye Movement Sleep Behavior Disorder in Parkinson’s Disease

**DOI:** 10.3389/fnins.2022.832550

**Published:** 2022-03-03

**Authors:** Nan-nan Yang, Shu-shan Sang, Tao Peng, Hong lu

**Affiliations:** ^1^Department of Neurology, The First Affiliated Hospital of Zhengzhou University, Zhengzhou, China; ^2^Department of Otolaryngology, The First Affiliated Hospital of Zhengzhou University, Zhengzhou, China

**Keywords:** PPMI, Parkinson’s disease, REM sleep behavior disorder, *SNCA* rs3910105, alpha-synuclein

## Abstract

**Background and Purpose:**

Rapid eye movement (REM) Rapid eye movement sleep behavior disorder (RBD) is a common non-motor symptom of PD. However, the association between the *SNCA* rs3910105 genotype and RBD in Parkinson’s disease (PD) remains unclear.

**Methods:**

This study used Parkinson’s Progression Markers Initiative (PPMI) data and included 270 patients with newly diagnosed PD without RBD who were divided into *SNCA* rs3910105 C carriers (CC+CT; *n* = 187) and TT carriers (*n* = 83). They were followed up for 5 years to identify the development of RBD. To investigate the influence of cerebrospinal fluid (CSF) alpha-synuclein (α-syn) and β-amyloid 1–42 (Aβ_42_) in the association between rs3910105 and RBD, the patients were additionally classified into “high-level” and “low-level” groups using cutoff values for CSF α-syn and Aβ_42_ levels.

**Results:**

At baseline, the rs3910105 C allele group had lower CSF α-syn and Aβ_42_ levels than the TT group. During the 5.0-year follow-up, the rs3910105 C allele group had a higher incidence of RBD than the TT group. In the subgroup analyses, the effect of the rs3910105 C allele was not found in the “low-level” group. However, in the “high-level” group, the rs3910105 C allele independently increased the risk of RBD.

**Conclusion:**

The *SNCA* rs3910105 C allele might be a novel genetic risk factor for RBD development in PD, α-syn pathways might have a role in this association and more basic research would be needed to elucidate the mechanism in the future.

## Introduction

Parkinson’s disease (PD) is an age-related neurodegenerative disease with a wide range of motor and non-motor symptoms (Chaudhuri et al., 2006; Bloem et al., 2021). Rapid eye movement (REM) Rapid eye movement sleep behavior disorder (RBD) is a common non-motor symptom of PD that is characterized by the loss of normal muscle atonia during REM sleep and involves dream-enacting behavior (Dauvilliers et al., 2018; Miglis et al., 2021). PD patients have a prevalence of RBD symptomatology of approximately 40% (Romenets et al., 2012; Zhang et al., 2017; Diaconu et al., 2021). It has been suggested that older age and longer disease duration are risk factors for RBD in patients with PD (Lee et al., 2010; Romenets et al., 2012). Previous studies found that at baseline, cerebrospinal fluid (CSF) alpha-synuclein (α-syn) and CSF β-amyloid 1–42 (Aβ_42_) were significantly lower in PD subjects with RBD (Ba et al., 2018; Pagano et al., 2018). Given that CSF α-syn and Aβ_42_ levels inversely correlate with α-syn and Aβ pathology in the brain (Fagan et al., 2006; Wennström et al., 2013; Gold et al., 2021), this observation may support the notion that cerebral synucleinopathy or amyloidopathy contributes to the development of RBD.

The aggregation of α-syn, encoded by the *SNCA* gene, is central to the pathogenesis of PD (Deng and Yuan, 2014; Meade et al., 2019). The *SNCA* rs3910105 T/C polymorphism has been shown to alter the risk of developing PD (Goris et al., 2007; Nalls et al., 2014; Campêlo and Silva, 2017). Previous studies showed that rs3910105 was a significant predictor of changes in Aβ_42_, which has been shown to be relevant in PD neurodegeneration (Mollenhauer et al., 2017). Another study revealed that rs3910105 was associated with the annual percentage change in dopamine transporter (DAT) availability, indicating that the rs3910105 single nucleotide polymorphisms (SNPs) might affect the progression of PD (Shin et al., 2019).

However, the relationship between the *SNCA* rs3910105 C allele and RBD development in PD patients remains unclear. Therefore, the aim of this study was to examine the associations between the *SNCA* rs3910105, CSF α-syn and Aβ_42_ levels and the development of RBD in a large group of patients with early PD.

## Materials and Methods

### Study Design and Patients

The data were obtained from the Parkinson’s Progression Markers Initiative (PPMI) database. The PPMI study is an ongoing international, multicenter, cohort study to identify biomarkers of progression in PD. The study protocol and methodology are available on the PPMI website^1^ and have been published elsewhere (Marek et al., 2018). Patients were recruited between 2010 and 2015 through strict inclusion/exclusion criteria, as previously described.

We downloaded data on PD patients from the PPMI database in December 2020. For this analysis, data up to 5 years of follow-up were included. We excluded patients who had RBD at the baseline visit or were functionally dependent at or were lost to follow-up in the first year following enrollment. Finally, a total of 270 patients were involved in this analysis.

### *SNCA rs3910105* Genotyping

DNA was extracted from the whole blood of the patients according to the study protocol as described in the PPMI biologics manual. All sample were genotyped using the Illumina NeuroX array following the manufacturer’s protocol. The Genotyping Analysis Module within Genome Studio was used to analyze data (Nalls et al., 2016).

### Ascertainment of Rapid Eye Movement Sleep Behavior Disorder

The presence of probable RBD was defined based on the RBD Screening Questionnaire (RBDSQ). The RBDSQ comprises 10 items investigating the patient’s sleep behavior. The total score is a maximum of 13 points. Only dichotomic responses (“yes” or “no”) were allowed. Dream frequency, content, and their relation to nocturnal movements and behavior were assessed by items 1–4. Item 5 evaluated the presence of self-injuries and injuries to the bed partner while asleep. Item 6 explored nocturnal vocalization, limb movements, complex movements, and objects that fall down around the bed. Items 7 and 8 assessed nocturnal awakenings. Items 9 and 10 investigated the presence of global disturbed sleep and any other neurologic disorder. As previously reported (Berg et al., 2021), an RBDSQ score of 5 was used as a cutoff to differentiate patients with RBD (RBDSQ ≥5) from those without RBD (RBDSQ <5). When using RBDSQ, a better sensitivity is reached by using a cut off score of 1 for item 6 (including 6.1–6.4), sensitivity increases to 0.74 at a reasonable specificity of 0.70 in the PD cohort. (PPMI cohort) (Halsband et al., 2018; Halsband et al., 2018), so RBDSQ ≥5 and item6 ≥1 combined as the cutoff for PD patient with RBD.

### Cerebrospinal Fluid Sample Collection and Assessments

CSF was collected and analyzed for α-syn, total tau (t-tau), tau phosphorylated at Thr181 (p-tau), and Aβ_42_ as previously described (Pagano et al., 2016, 2018). Briefly, CSF was collected using standardized lumbar puncture procedures. CSF was collected into siliconized polypropylene tubes, and the first 1 to 2 mL of CSF was sent to the site’s local laboratory for routine testing for cell count, total protein level, and glucose level. An additional 15 to 20 mL of CSF was transferred into15-mL conical polypropylene tubes at room temperature, mixed gently, centrifuged at 2000 *g* for 10 min at room temperature, and transferred into 1.5-mL precooled siliconized polypropylene aliquot tubes followed by immediate freezing on dry ice. The frozen aliquots of CSF were shipped overnight to the PPMI Biorepository Core laboratories on dry ice and then thawed, aliquoted into 0.5-mL siliconized polypropylene tubes, refrozen once, and stored at −80°C. Measurements of CSF Ab42, tau, and p-tau were made using the xMAP-Luminex platform with INNOBIA AlzBio3 immunoassay kit–based reagents (Fujirebio-Innogenetics, Ghent, Belgium) at Penn. Commercially available sandwich type immunoassay kits (BioLegend; formerly Covance) were used to analyze CSF a-syn and CSF hemoglobin levels.

Further information can be found in the PPMI biologics manual (see text footnote 1).

### Clinical Evaluation

Motor symptom severity was assessed with the Movement Disorder Society–sponsored revision of the Unified Parkinson’s Disease Rating Scale (MDS-UPDRS) Part III and Hoehn and Yahr (H&Y) scale, and non-motor symptom burden was assessed with MDS-UPDRS Part I. Global cognitive status was assessed using the Montreal Cognitive Assessment (MoCA). Domain-specific neuropsychological performance was measured using 7 standardized instruments. Verbal memory was assessed by the Hopkins Verbal Learning Test, with measures of immediate recall, delayed recognition false alarms, and delayed recognition hits. Verbal fluency was assessed by the semantic fluency test. Processing speed was assessed by the Symbol Digit Modalities Test. Visuospatial abilities were assessed by the Benton Judgment of Line Orientation Test. Executive functioning was assessed by the letter-number sequencing test. Depressive symptoms were assessed with the short version of the Geriatric Depression Scale (15-item GDS). Anxiety was assessed with Spielberger’s State-Trait Anxiety Inventory (STAI). Autonomic dysfunction was assessed with the Scales for Outcomes in Parkinson’s Disease–Autonomic (SCOPA-AUT). Sleep disturbances were assessed with the Epworth Sleepiness Scale. The Questionnaire for Impulsive Compulsive Disorders in PD was used to assess impulsive-compulsive disorders and other compulsive behaviors. Olfactory dysfunction was measured by the University of Pennsylvania Smell Identification Test (UPSIT). Clinical evaluation was performed in the “off” treatment state.

### Statistical Analysis

Calculations were performed with SPSS 25.0 (SPSS Inc., Chicago, IL, United States) and R version 3.6.2 (R Foundation for Statistical Computing, Vienna, Austria). Data are presented as the mean ± standard deviation. Data normality was assessed with the use of the Shapiro-Wilk test. Baseline characteristics were compared with Student’s *t*-tests continuous variables and chi-square tests or Fisher’s exact tests for categorical variables, as appropriate. We used the Kaplan-Meier method to compare the cumulative incidence of RBD between the groups. Cox proportional hazards regression models were used for an adjusted analysis of the relationship of the SNCA rs3910105 allele with RBD, and hazard ratios (HRs) and 95% confidence intervals (CIs) were calculated. All baseline variables with *p*-values less than 0.2 in the univariable Cox models were included in subsequent multivariable Cox models with a backward elimination procedure (*p*-value removal = 0.1). All *p*-values were two-sided, and a *p*-value of less than 0.05 was regarded as statistically significant. As for the cut off in the subgroup analysis, we used an optimum cutoff value for the CSF α-syn and Aβ_42_ levels using the R package “MaxStat,” which selects the cutoff value producing the maximum log-rank score. To increase stability of our findings, we also used the 25th and 50th percentile cutoff values of the CSF Aβ_42_ levels. All *p-*values were two-sided, and a *p*-value of less than 0.05 was regarded statistically significant.

## Results

### Demographic and Clinical Features

Among the 270 patients [age(61.5 ± 9.7); men, (66%)] included in this study, 186 (68.8%) were SNCA rs3910105 C carriers, and 84 (31.2%) were TT carriers. The baseline characteristics of the two groups are depicted in Table 1. There were no significant differences between-group in age, sex, disease duration, age at onset or MDS-UPDRS motor, tremor, postural instability and gait difficulties (PIGD), MoCA, GDS, or STAI scores.

**TABLE 1:** Baseline characteristics in the rs3910105 C allele group and TT groups.

Variables	Total patients *N* = 270 Mean(SD)	CC/CT group *N* = 186 Mean(SD)	TT group *N* = 84 Mean(SD)	*P*-value
Age, yeas	61.5 (9.7)	61.46 (10.16)	62.10 (9.35)	0.6225
Sex, male(%)	178 (66%)	120 (64%)	58 (69%)	0.467
Disease duration, months	6.7 (6.6)	7.2 (7.1)	5.9 (5.3)	0.099
Age at onset, years	59.56 (10.12)	60.32 (9.22)	59.27 (10.94)	0.44
MDS-UPDRS motor score	20.0 (9.19)	20.13 (9.61)	19.90 (8.269)	0.89
MDS-UPDRS score	30.01 (11.23)	30.24 (13.02)	29.45 (10.69)	0.87
Tremor score	4.38 (3.15)	4.42 (3.35)	4.53 (3.364)	0.81
Rigidity score	3.54 (2.70)	3.39 (2.73)	3.75 (2.32)	0.23
UPSIT Score	23.46 (8.60)	23.83 (8.74)	24.13 (7.60)	0.78
STAI score	62.96 (17.12)	63.41 (17.45)	61.96 (16.40)	0.52
GDS score	2.1 (2.49)	2.21 (2.57)	1.845 (2.32)	0.26
QUIP score	0.27 (0.62)	0.27 (0.62)	0.27 (0.63)	0.98
ESS score	5.68 (3.42)	5.69 (3.50)	5.65 (3.28)	0.92
SCOPA-AUT score	8.41 (5.58)	8.48 (5.50)	8.25 (5.77)	0.81
BJLO score	13.00 (2.0)	13.06 (1.89)	12.86 (2.12)	0.734
MoCA score	27.15 (2.26)	27.08 (2.355)	27.28 (2.223)	0.78
RBD score	2.42 (1.16)	2.42 (1.18)	2.44 (1.11)	0.92
Caudate DAT uptake	2.0 (0.57)	2.1 (0.61)	2.10 (0.60)	0.90
Putamen DAT uptake	0.96 (0.49)	0.97 (0.49)	0.94 (0.48)	0.61

**FIGURE 1:**
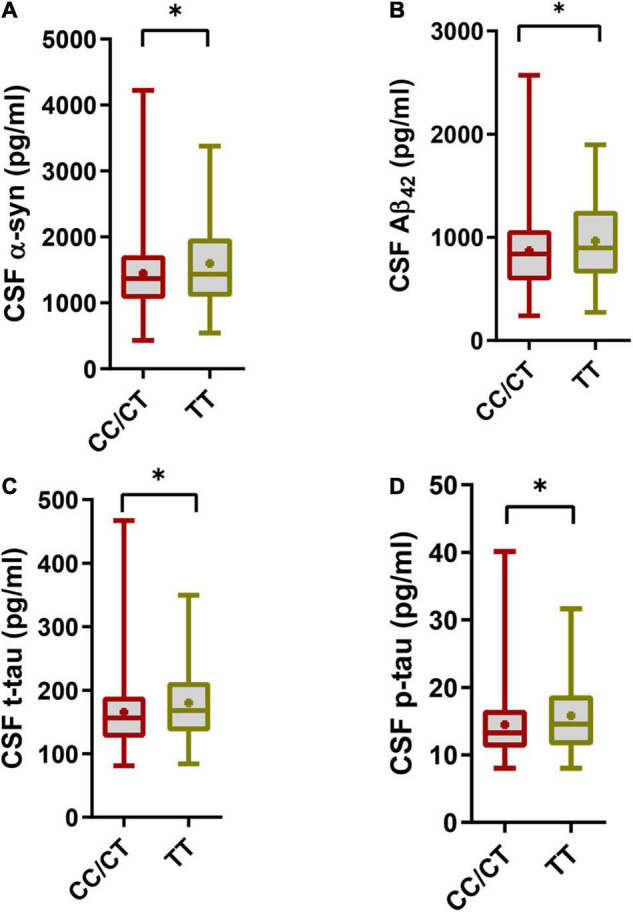
Cerebrospinal fluid (CSF) pathology in the SNCA rs3910105 C allele group and TT group. **(A)** Difference in CSF α-syn levels between the rs3910105 C allele and TT groups [Unpaired *t*-test(two-tailed), *t* = 2.38, *p* = 0.019]; **(B)** differences in CSF Aβ_42_ levels between the rs3910105 C allele and TT groups [Unpaired *t*-test(two-tailed), *t* = 2.43, *p* = 0.015]; **(C)** differences in CSF t-tau levels between the rs3910105 C allele and TT groups [Unpaired *t-*test(two-tailed), *t* = 2.47, *p* = 0.012]; **(D)** differences in CSF p-tau levels between the rs3910105 C allele and TT groups [Unpaired *t*-test(two-tailed), *t* = 2.31, *p* = 0.02]. *P < 0.05.

However, the SNCA rs3910105 C allele group had lower CSF α-syn levels (1448 ± 592.6 pg/ml vs. 1601 ± 668.5 pg/ml; *p* = 0.019) (Figure 1A), lower CSF Aβ_42_ levels (870.4 ± 370 pg/ml vs. 965.7 ± 377.3 pg/ml; *p* = 0.015) (Figure 1B), lower CSF t-tau levels (165.3 ± 55.43 pg/ml vs. 180 ± 59.82 pg/ml; *p* = 0.012) (Figure 1C) and lower CSF p-tau levels (14.48 ± 5.124 vs. 15.80 ± 5.659, *p* = 0.02) (Figure 1D) than the TT group.

### Rs3910105 and the Development of Rapid Eye Movement Sleep Behavior Disorder

**FIGURE 2:**
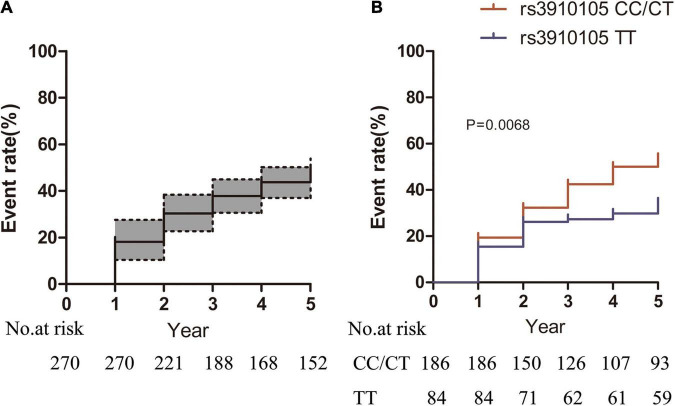
Kaplan-Meier estimates showing the cumulative risk of RBD in **(A)** the total sample of patients and **(B)** the rs3910105 C carrier group and TT group. In panel **(A)**, dashed lines represent the 95% confidence interval range. In panel **(B)**, the red line indicates the rs3910105 C allele group, and the blue line indicates the TT group.

During the follow-up, a total of 129 (47.7%) patients developed RBD, and the cumulative incidence of RBD was 18.1, 37.7, and 43.7% at the 1-, 2- and 4-year follow-ups, respectively (Figure 2A). At baseline, those patients who later developed RBD had a higher prevalence of the *SNCA* rs3910105 C allele, higher MDS-UPDRS total and motor scores and lower CSF Aβ_42_ levels than those who did not develop RBD (Table 2). There were 100 patients (53.4%) in the rs3910105 C allele group and 29 patients (34.5%) in the TT group who developed RBD during the follow-up period. Kaplan-Meier estimates showed that the rs3910105 C allele group had a significantly higher incidence of RBD than the TT group (*p* = 0.0068) (Figure 2B).

**TABLE 2:** Baseline characteristics of PD patients who later developed RBD or without RBD during.

Variables	Total patients *N* = 270 Mean(SD)	RBD *N* = 129 Mean(SD)	NO RBD *N* = 141 Mean(SD)	*P*-value
rs3910105 C allele	186 (84)	100 (29)	86 (55)	0.003
Age, yeas	61.5 (9.7)	61.50 (10.39)	61.86 (9.49)	0.76
Sex, male(%)	178 (66%)	85 (65.9%)	93 (65.9%)	0.99
Disease duration, months	6.7 (6.6)	7.122 (7.2)	6.69 (6.0)	0.61
Age at onset, years	59.56 (10.12)	59.43 (11.02)	59.83 (9.87)	0.75
MDS-UPDRS score	29.99 (11.19)	32.34 (13.74)	27.79 (10.51)	0.0024
MDS-UPDRS motor score	20.0 (9.19)	21.00 (9.737)	19.01 (8.267)	0.07
Tremor score	4.38 (3.15)	4.42 (3.45)	4.57 (3.28)	0.71
Rigidity score	3.54 (2.70)	3.79 (2.75)	3.22 (2.47)	0.07
CSF α-syn	1482 (591.3)	1415 (618.8)	1523 (566.2)	0.12
CSF Aβ_42_	893.3 (367.1)	847.7 (324.2)	926.3 (373.3)	0.07
CSF total tau	166.4 (54.41)	160.6 (55.60)	171.9 (53.06)	0.09
CSF p-tau	14.55 (5.04)	14.24 (5.19)	14.85 (4.919)	0.34

The results of the Cox regression analyses are shown in Table 3. In the univariable Cox regression analyses and multivariable Cox analyses, the rs3910105 C allele and MDS-UPDRS scores were significantly related to RBD.

**TABLE 3:** Results of the Cox regression analyses for the predictors of RBD.

	Univariable analysis		Multivariable analysis	
Variables	HR(95% CI)	*P*-value	HR(95% CI)	*P*-value
rs3910105 C allele	1.698 (1.122–2.569)	0.012	1.688 (1.115–2.555)	0.013
Age	0.999 (0.982–1.017)	0.915	–	–
Male sex	1.003 (0.836–1.203)	0.992	–	–
Disease duration	0.998 (0.996–1.001)	0.998	–	–
MDS-UPDRS score	1.026 (1.012–1.041)	<0.001	1.019 (1.004–1.035)	0.011
Tremor score	1.006 (0.988–1.025)	0.493	–	–
Rigidity score	1.065 (0.999–1.136)	0.052	–	–
CSF α-syn	1.012 (0.978–1.123)	0.216	–	–
CSF Aβ_42_	1.015 (0.968–1.125)	0.202	–	–
CSF total tau	0.998 (0.996–1.001)	0.199	–	–
CSF p-tau	0.969 (0.957–1.002)	0.12	–	–

### Subgroup Analyses

Although the baseline levels of CSF α-syn and CSF Aβ_42_ could not be related to the future incidence of RBD based on the Cox regression analysis data, as shown in Figure 1, we knew that CSF α-syn and CSF Aβ_42_ levels were much lower in the rs3910105 C allele group than in the TT group. Since the RBD development rate was much higher in the C allele group than in the TT group, we speculated that lower baseline CSF α-syn and CSF Aβ_42_ might also contribute to the higher incidence of RBD. We divided the patients into two groups based on the CSF α-syn and CSF Aβ_42_ levels.

**FIGURE 3:**
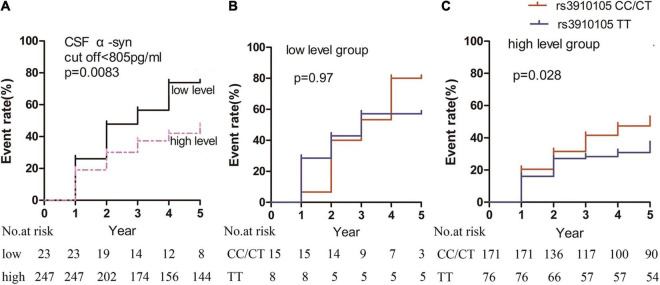
Kaplan-Meier estimates showing the cumulative risk of RBD based on α-syn levels in the **(A)** “low-level” and “high-level” groups, **(B)** rs3910105 C carriers and non-carriers in the “low-level” group, and **(C)** rs3910105 C carriers and non-carriers in the “high-level” group. In panel **(A)**, the black line indicates the “low-level” group, and the dashed purple line indicates the “high-level” group. In panels **(B,C)**, red lines indicate rs3910105 C carriers, and blue lines indicate non-carriers.

The cutoff value of CSF α-syn in relation to RBD for producing the maximum log rank score was 805 pg/ml. When the patients were divided by this cutoff value, the “low-level” group (17/23 patients [74%]) had a significantly higher incidence of RBD than the “high-level” group (106/247 patients [43%]; *p* = 0.0083) (Figure 3A).

Furthermore, in the “low-level” group, there was no significant difference in the development of RBD between the rs3910105 C carriers and TT group (*p* = 0.97) (Figure 3B). However, in the “high-level” group, the cumulative incidence of RBD was significantly higher in the rs3910105 C carriers than in the non-carriers (*p* = 0.028) (Figure 3C).

**FIGURE 4:**
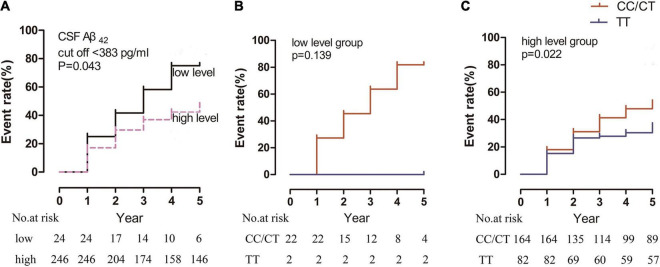
Kaplan-Meier estimates showing the cumulative risk of RBD based on Aβ_42_ levels in the **(A)** “low-level” and “high-level” groups, **(B)** rs3910105 C and non-carriers in the “low-level” group, and **(C)** rs3910105 C carriers and non-carriers in the “high-level” group. In panel **(A)**, the black line indicates the “low-level” group, and the dashed purple line indicates the “high-level” group. In panels **(B,C)**, red lines indicate rs3910105 C carriers, and blue lines indicate non-carriers.

Regarding CSF Aβ_42_ levels, the cutoff value of CSF Aβ_42_ in relation to RBD for producing the maximum log rank score was 385 pg/ml. When the patients were divided by this cutoff value, the “low-level” group (18/24 patients [75%]) had a significantly higher incidence of RBD than the “high-level” group (104/246 patients [42%]; *p* = 0.043) (Figure 4A).

In the “low-level” group, there was no significant difference in the development of RBD between the rs3910105 C carriers and TT group (*p* = 0.97) (Figure 4B). However, in the “high-level” group, the cumulative incidence of RBD was significantly higher in the rs3910105 C carriers than in the non-carriers (*p* = 0.022) (Figure 4C).

## Discussion

This study investigated the relationship of the SNCA rs3910105 C allele with the development of RBD in patients with early PD using the PPMI cohort. Our results showed that the *SNCA* rs3910105 C allele was associated with faster development of RBD in PD patients, suggesting that the *SNCA* rs3910105 C allele was a novel genetic risk factor for RBD. To our knowledge, this was the first study to demonstrate the association of *SNCA* rs3910105 and RBD development in PD patients.

Studies have shown that rs3910105 was associated with CSF Aβ_42_ levels (Mollenhauer et al., 2017), which was consistent with our results that CSF pathology (including α-syn, Aβ_42_, t-tau and p-tau) in the *SNCA* rs3910105 C allele group was much lower than that in the TT group. Furthermore, studies have found that CSF α-syn and Aβ_42_ were significantly lower in PD subjects with RBD (Ba et al., 2018; Pagano et al., 2018). Therefore, we speculated that the *SNCA* rs3910105 C allele group might have a higher incidence of RBD development due to the C allele group’s lower CSF pathology, which may contribute to the development of RBD. The Cox regression analysis results confirmed our hypothesis. Based on the results, the *SNCA* rs3910105 C allele and MDS-UPDRS total scores were related to RBD incidence. However, none of the CSF pathologies contributed to RBD development according to our Cox model, which might be related to a limited number of patients. Since there came a trend that the baseline CSF pathology levels (α-syn and Aβ_42_) were lower in PD patients who later developed RBD, we did further subgroup analysis that divided the PD patients into low and high groups based on the baseline CSF pathology levels (α-syn and Aβ_42_), and it turned out that the “low-level” group did have a significantly higher incidence of RBD than the “high-level” group. However we found the effect of the rs3910105 C allele was not in the “low-level” group, which was possible considering the limited number of this group and a low CSF pathology baseline level that they already had, the rs3910105 C allele effect to keep the low level of CSF pathology and then increase the risk of RBD development as PD progress might not work. While in the “high-level” group, the effect was still there, the rs3910105 C allele prevalence contributed to a lower CSF α-syn and Aβ_42_ both at baseline and during the follow up study, thus increasing the risk of RBD during disease progress. which indirectly showed an association between CSF α-syn and Aβ_42_ levels and RBD incidence.

Rs3910105 is an intronic variant in the *SNCA* gene and is located in the CpG island of *SNCA* (Tagliafierro and Chiba-Falek, 2016; Funahashi et al., 2017). The *SNCA* gene encodes α-syn, which consists of 140 amino acid residues and is involved in the etiology of PD and other neurodegenerative diseases (Attwood et al., 2002; Mata et al., 2010). Since the CpG island methylation level was able to regulate gene expression (Attwood et al., 2002; Moore et al., 2013), the rs3910105 SNP somehow regulates different *SNCA* transcript targets. A recent transcript-level linear modeling study based on the PPMI database showed that the C allele of rs3910105 was able to upregulate *SNCA* gene expression (Koks et al., 2021). Furthermore, another study found that the rs3910105 genotype showed negative correlations with DAT availability in the putamen with *SNCA* transcripts (Shin et al., 2019), indicating that the rs3910105 C allele was able to impact α-syn expression and accumulation and even the progression of PD development.

**FIGURE 5:**
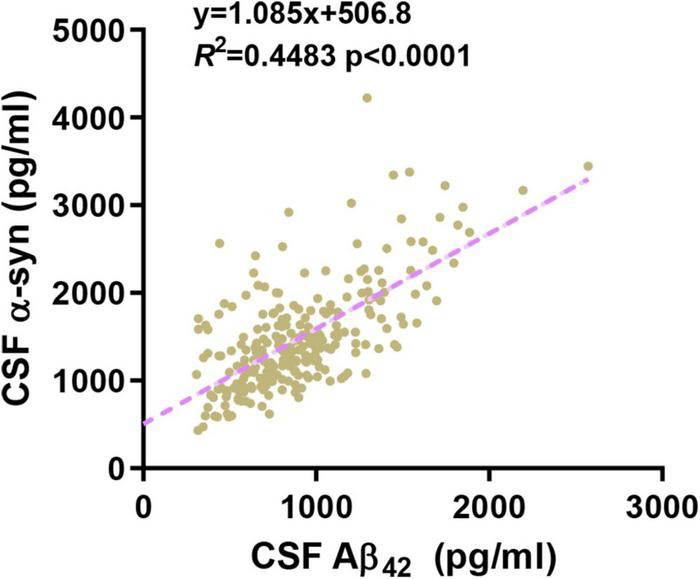
Cerebrospinal fluid (CSF) α-syn levels correlated with Aβ_42_ levels.

Previous studies have demonstrated that RBD was a strong predictor for the development of synucleinopathy, most frequently PD or dementia with Lewy bodies (DLB) (Högl et al., 2018). However, the incidence of RBD development in early PD patients and the underlying pathogenesis have not received much attention. In our longitudinal observational study, we shed light on the role of rs3910105 in RBD development in early PD patients, which might occur through the contribution of cerebral synucleinopathy. Even though the Cox model did not provide a direct correlation between CSF pathology and RBD development in PD patients, we obtained more information through a subgroup analysis in which a lower level of CSF α-syn was related to a higher incidence of RBD. As we previously cited, rs3910105 was a predictor of changes in Aβ_42_, but the underlying mechanism remains unknown. Studies on the impact of rs3910105 on Aβ_42_ are limited, and elucidation of this relationship needs more investigation. Studies have shown that CSF α-syn is positively correlated with Aβ_42_ in PD participants (Buddhala et al., 2015). We also performed correlation analysis in our study and found that CSF α-syn correlated with Aβ_42_ (*R*^2^ = 0.44) (Figure 5), suggesting a pathophysiologic connection between the metabolism of these proteins in PD.

There were some limitations to note in this study. First, the presence of RBD was based on results from the RBDSQ, while a definite diagnosis of RBD requires polysomnography (PSG). Because PSG is expensive and requires a skilled examiner, the RBDSQ is used more frequently in large cohort follow-up studies. In this context, the RBD data in the current study might have been misestimated. Second, even though using the subgroup analysis, we indirectly showed an association between baseline CSF α-syn and Aβ_42_ levels and RBD incidence, but the Cox model did not work. We could only speculate this association may related with contributions made by rs3910105 C allele.

In conclusion, our results suggest that the rs3910105 C allele is associated with an increased risk of RBD development in PD, and α-syn pathways might have a role in this association Further studies need to be conducted to validate our findings and to reveal the precise mechanisms underlying the influence of the rs3910105 C allele in the development of RBD in patients with PD.

## Data Availability Statement

The datasets presented in this study can be found in online repositories. The names of the repository/repositories and accession number(s) can be found in the article/supplementary material.

## Ethics Statement

The studies involving human participants were reviewed and approved by Parkinson’s Progression Markers Initiative (PPMI). The patients/participants provided their written informed consent to participate in this study.

## Author Contributions

N-NY conceived and designed the project and prepared the writing of the first draft. S-SS designed and executed most of the statistical analysis and reviewed the first draft. TP executed some statistical analysis and prepared the figures and tables. Hl conceived and designed the project, make the critique, and reviewed the final draft. All authors contributed to the article and approved the submitted version.

## Conflict of Interest

The authors declare that the research was conducted in the absence of any commercial or financial relationships that could be construed as a potential conflict of interest.

## Publisher’s Note

All claims expressed in this article are solely those of the authors and do not necessarily represent those of their affiliated organizations, or those of the publisher, the editors and the reviewers. Any product that may be evaluated in this article, or claim that may be made by its manufacturer, is not guaranteed or endorsed by the publisher.

## Footnotes

1
http://ppmi-info.org

## References

[B1] Attwood J. Yung R. Richardson B. (2002). DNA methylation and the regulation of gene transcription. *Cell. Mol. Life Sci. CMLS* 59 241–257. 10.1007/s00018-002-8420-z 11915942 PMC11146104

[B2] Ba M. Yu G. Kong M. Liang H. Yu L. (2018). CSF Aβ level is associated with cognitive decline in early Parkinson’s disease with rapid eye movement sleep behavior disorder. *Trans. Neurodegenerat.* 7:22. 10.1186/s40035-018-0129-5 30338062 PMC6174574

[B3] Berg D. Borghammer P. Fereshtehnejad S. Heinzel S. Horsager J. Schaeffer E. (2021). Prodromal parkinson disease subtypes - key to understanding heterogeneity. *Nat. Rev. Neurol.* 17 349–361. 10.1038/s41582-021-00486-9 33879872

[B4] Bloem B. Okun M. Klein C. (2021). Parkinson’s disease. *Lancet (London, England)* 397 2284–2303. 10.1016/s0140-6736(21)00218-x 33848468

[B5] Buddhala C. Campbell M. Perlmutter J. Kotzbauer P. (2015). Correlation between decreased CSF α-synuclein and Aβ_1–42_ in parkinson disease. *Neurobiol. Aging* 36 476–484. 10.1016/j.neurobiolaging.2014.07.043 25212463 PMC4268043

[B6] Campêlo C. Silva R. (2017). Genetic variants in SNCA and the risk of sporadic Parkinson’s disease and clinical outcomes: a review. *Parkinson’s Dis.* 2017:4318416. 10.1155/2017/4318416 28781905 PMC5525082

[B7] Chaudhuri K. Martinez-Martin P. Schapira A. Stocchi F. Sethi K. Odin P. (2006). International multicenter pilot study of the first comprehensive self-completed nonmotor symptoms questionnaire for Parkinson’s disease: the NMSQuest study. *Movement Dis. Off. J. Movement Dis. Soc.* 21 916–923. 10.1002/mds.20844 16547944

[B8] Dauvilliers Y. Schenck C. H. Postuma R. B. Iranzo A. Luppi P. H. Plazzi G. (2018). REM sleep behaviour disorder. *Nat. Rev. Dis. Primers* 4:19. 10.1038/s41572-018-0016-5 30166532

[B9] Deng H. Yuan L. (2014). Genetic variants and animal models in SNCA and parkinson disease. *Ageing Res. Rev.* 15 161–176. 10.1016/j.arr.2014.04.002 24768741

[B10] Diaconu Ş Falup-Pecurariu O. Ţînţ D. Falup-Pecurariu C. (2021). REM sleep behaviour disorder in Parkinson’s disease (review). *Exp. Ther. Med.* 22:812. 10.3892/etm.2021.10244 34131435 PMC8193212

[B11] Fagan A. M. Mintun M. A. Mach R. H. Lee S. Y. Dence C. S. Shah A. R. (2006). Inverse relation between in vivo amyloid imaging load and cerebrospinal fluid Abeta42 in humans. *Ann. Neurol.* 59 512–519. 10.1002/ana.20730 16372280

[B12] Funahashi Y. Yoshino Y. Yamazaki K. Mori Y. Mori T. Ozaki Y. (2017). DNA methylation changes at SNCA intron 1 in patients with dementia with lewy bodies. *Psychiatry Clin. Neurosci.* 71 28–35. 10.1111/pcn.12462 27685250

[B13] Gold B. Shao X. Sudduth T. Jicha G. Wilcock D. Seago E. (2021). Water exchange rate across the blood-brain barrier is associated with CSF amyloid-β 42 in healthy older adults. *Alzheimer’s Dementia J. Alzheimer’s Assoc.* 17 2020–2029. 10.1002/alz.12357 33949773 PMC8717840

[B14] Goris A. Williams-Gray C. Clark G. Foltynie T. Lewis S. Brown J. (2007). Tau and alpha-synuclein in susceptibility to, and dementia in, Parkinson’s disease. *Ann. Neurol.* 62 145–153. 10.1002/ana.21192 17683088

[B15] Halsband C. Zapf A. Sixel-Doring F. Trenkwalder C. Mollenhauer B. (2018). The REM sleep behavior disorder screening questionnaire is not valid in de novo Parkinson’s disease. *Mov. Disord Clin. Pract.* 5 171–176. 10.1002/mdc3.12591 30009211 PMC6033034

[B16] Högl B. Stefani A. Videnovic A. (2018). Idiopathic REM sleep behaviour disorder and neurodegeneration - an update. *Nat. Rev. Neurol.* 14 40–55. 10.1038/nrneurol.2017.157 29170501

[B17] Koks S. Pfaff A. Bubb V. Quinn J. (2021). Transcript variants of genes involved in neurodegeneration are differentially regulated by the APOE and MAPT haplotypes. *Genes* 12:423. 10.3390/genes12030423 33804213 PMC7999745

[B18] Lee J. Kim K. Shin H. Sohn Y. (2010). Factors related to clinically probable REM sleep behavior disorder in parkinson disease. *Parkinsonism Related Dis.* 16 105–108. 10.1016/j.parkreldis.2009.08.005 19716333

[B19] Marek K. Chowdhury S. Siderowf A. Lasch S. Coffey C. Caspell-Garcia C. (2018). The Parkinson’s progression markers initiative (PPMI) - establishing a PD biomarker cohort. *Ann. Clin. Trans. Neurol.* 5 1460–1477. 10.1002/acn3.644 30564614 PMC6292383

[B20] Mata I. F. Shi M. Agarwal P. Chung K. A. Edwards K. L. Factor S. A. (2010). SNCA variant associated with parkinson disease and plasma alpha-synuclein level. *Arch. Neurol.* 67 1350–1356. 10.1001/archneurol.2010.279 21060011 PMC3010848

[B21] Meade R. Fairlie D. Mason J. (2019). Alpha-synuclein structure and Parkinson’s disease - lessons and emerging principles. *Mol. Neurodege.* 14:29. 10.1186/s13024-019-0329-1 31331359 PMC6647174

[B22] Miglis M. Adler C. Antelmi E. Arnaldi D. Baldelli L. Boeve B. (2021). Biomarkers of conversion to α-synucleinopathy in isolated rapid-eye-movement sleep behaviour disorder. *Lancet Neurol.* 20 671–684. 10.1016/s1474-4422(21)00176-9 34302789 PMC8600613

[B23] Mollenhauer B. Caspell-Garcia C. Coffey C. Taylor P. Shaw L. Trojanowski J. (2017). Longitudinal CSF biomarkers in patients with early parkinson disease and healthy controls. *Neurology* 89 1959–1969. 10.1212/wnl.0000000000004609 29030452 PMC5679418

[B24] Moore L. Le T. Fan G. (2013). DNA methylation and its basic function. *Neuropsychopharmacol. Off. Publi. Am. College Neuropsychopharmacol.* 38 23–38. 10.1038/npp.2012.112 22781841 PMC3521964

[B25] Nalls M. Keller M. Hernandez D. Chen L. Stone D. Singleton A. (2016). Baseline genetic associations in the Parkinson’s progression markers initiative (PPMI). *Mov. Dis. Off. J. Mov. Dis. Soc.* 31 79–85. 10.1002/mds.26374 26268663 PMC4724309

[B26] Nalls M. Pankratz N. Lill C. Do C. Hernandez D. Saad M. (2014). Large-scale meta-analysis of genome-wide association data identifies six new risk loci for Parkinson’s disease. *Nat. Genet.* 46 989–993. 10.1038/ng.3043 25064009 PMC4146673

[B27] Pagano G. De Micco R. Yousaf T. Wilson H. Chandra A. Politis M. (2018). REM behavior disorder predicts motor progression and cognitive decline in parkinson disease. *Neurology* 91 e894–e905. 10.1212/wnl.0000000000006134 30089615

[B28] Pagano G. Ferrara N. Brooks D. Pavese N. (2016). Age at onset and parkinson disease phenotype. *Neurology* 86 1400–1407. 10.1212/wnl.0000000000002461 26865518 PMC4831034

[B29] Romenets S. Gagnon J. Latreille V. Panniset M. Chouinard S. Montplaisir J. (2012). Rapid eye movement sleep behavior disorder and subtypes of Parkinson’s disease. *Mov. Dis. Off. J. Mov. Dis. Soc.* 27 996–1003. 10.1002/mds.25086 22733427

[B30] Shin S. Kim K. Lee J. Kim E. Kim S. Kim I. (2019). Effect of single-nucleotide polymorphisms on decline of dopamine transporter availability in Parkinson’s disease. *J. Clin. Neurol. (Seoul, Korea)* 15 102–107. 10.3988/jcn.2019.15.1.102 30618224 PMC6325373

[B31] Tagliafierro L. Chiba-Falek O. (2016). Up-regulation of SNCA gene expression: implications to synucleinopathies. *Neurogenetics* 17 145–157. 10.1007/s10048-016-0478-0 26948950 PMC4907864

[B32] Wennström M. Surova Y. Hall S. Nilsson C. Minthon L. Boström F. (2013). Low CSF levels of both α-synuclein and the α-synuclein cleaving enzyme neurosin in patients with synucleinopathy. *PLoS One* 8:e53250. 10.1371/journal.pone.0053250 23308173 PMC3540093

[B33] Zhang J. Xu C. Liu J. (2017). Meta-analysis on the prevalence of REM sleep behavior disorder symptoms in Parkinson’s disease. *BMC Neurol.* 17:23. 10.1186/s12883-017-0795-4 28160778 PMC5292147

